# Incorporation of quantitative MRI in a model to predict temporal lobe epilepsy surgery outcome

**DOI:** 10.1093/braincomms/fcab164

**Published:** 2021-07-16

**Authors:** Marcia Morita-Sherman, Manshi Li, Boney Joseph, Clarissa Yasuda, Deborah Vegh, Brunno Machado De Campos, Marina K M Alvim, Shreya Louis, William Bingaman, Imad Najm, Stephen Jones, Xiaofeng Wang, Ingmar Blümcke, Benjamin H Brinkmann, Gregory Worrell, Fernando Cendes, Lara Jehi

**Affiliations:** 1 Department of Neurology, Epilepsy Center, Cleveland Clinic, Cleveland, OH, USA; 2 Department of Quantitative Health Sciences, Quantitative Health Sciences, Cleveland Clinic, Cleveland, OH, USA; 3 Department of Neurology, Mayo Clinic, Rochester, MN, USA; 4 Department of Neurology, University of Campinas, Campinas, Brazil; 5 Department of Neuropathology, University Hospitals, Erlangen, Germany

**Keywords:** quantitative MRI, prediction of epilepsy surgery outcome, temporal lobe epilepsy, volumetric measurements

## Abstract

Quantitative volumetric brain MRI measurement is important in research applications, but translating it into patient care is challenging. We explore the incorporation of clinical automated quantitative MRI measurements in statistical models predicting outcomes of surgery for temporal lobe epilepsy. Four hundred and thirty-five patients with drug-resistant epilepsy who underwent temporal lobe surgery at Cleveland Clinic, Mayo Clinic and University of Campinas were studied. We obtained volumetric measurements from the pre-operative T1-weighted MRI using NeuroQuant, a Food and Drug Administration approved software package. We created sets of statistical models to predict the probability of complete seizure-freedom or an Engel score of I at the last follow-up. The cohort was randomly split into training and testing sets, with a ratio of 7:3. Model discrimination was assessed using the concordance statistic (C-statistic). We compared four sets of models and selected the one with the highest concordance index. Volumetric differences in pre-surgical MRI located predominantly in the frontocentral and temporal regions were associated with poorer outcomes. The addition of volumetric measurements to the model with clinical variables alone increased the model’s C-statistic from 0.58 to 0.70 (right-sided surgery) and from 0.61 to 0.66 (left-sided surgery) for complete seizure freedom and from 0.62 to 0.67 (right-sided surgery) and from 0.68 to 0.73 (left-sided surgery) for an Engel I outcome score. 57% of patients with extra-temporal abnormalities were seizure-free at last follow-up, compared to 68% of those with no such abnormalities (*P*-value = 0.02). Adding quantitative MRI data increases the performance of a model developed to predict post-operative seizure outcomes. The distribution of the regions of interest included in the final model supports the notion that focal epilepsies are network disorders and that subtle cortical volume loss outside the surgical site influences seizure outcome.

## Teaser

Abbreviated summary

Morita-Sherman et al. demonstrate how sophisticated research imaging can cross the divide to clinical practice. They show how quantitative MRI can enhance epilepsy surgery outcome prediction. Volume changes in the structures highlighted were identified as outcome predictors, supporting the hypothesis that subtle cortical atrophy outside the resection influences seizure outcome.

## Graphical Abstract

**Graphical Abstract:**
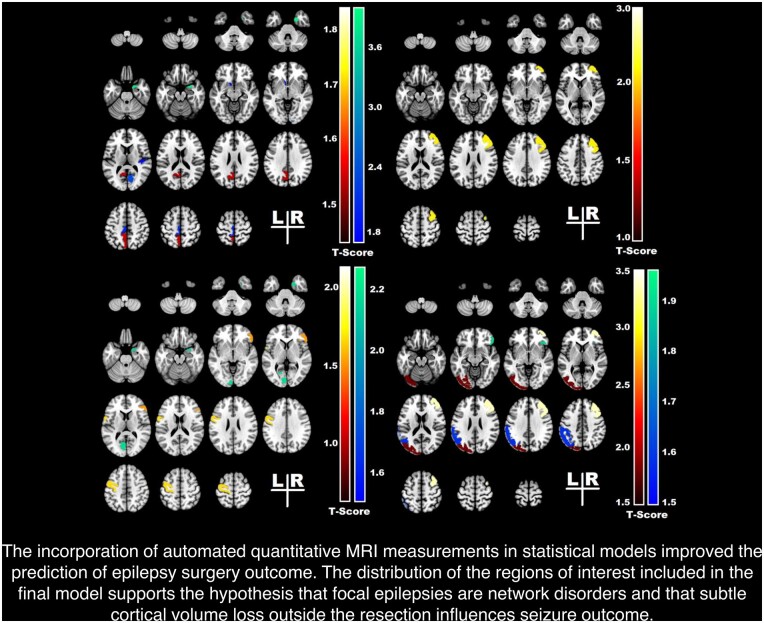

## INTRODUCTION

Surgery is usually the most effective treatment for drug-resistant focal epilepsies.^1^^,^^2^ Despite the extensive literature on epilepsy surgery, predicting the likelihood of postoperative seizure freedom for a given patient remains challenging.^3^^,^^4^ A nomogram to predict individual surgical outcomes using basic clinical variables^5^ was developed and validated by our group with modest accuracy (C-statistics of 0.6). Since brain MRI is a critical tool to localize the underlying epileptic lesion and the network of brain damage beyond the seizure focus,^6^^,^^7^ we hypothesize that the inclusion of quantitative MRI (qMRI) data may enhance the model’s accuracy.

Studies on volumetric measurements of the hippocampus enhanced our ability to detect signs of hippocampus atrophy on MRI and by doing so, revolutionized temporal lobe epilepsy (TLE) surgery. Volumetric measurement of brain structures is a long-standing research tool to detect MRI abnormalities that may not be readily identified by visual analysis.^8^ Studies on TLE surgery using different neuroimaging techniques have demonstrated that structural abnormalities in brain regions outside the surgical margins influence postoperative seizure outcomes.^8–11^ However, the translation of this imaging research knowledge into routine clinical practice has remained elusive, limiting its ultimate clinical impact.

The development of automatic segmentation algorithms enables volumetric brain measurement in clinical practice. We explore here the prognostic value of quantitative volumetric MRI measurements in the context of temporal lobe surgery, using NeuroQuant, an Food and Drug Administration approved software (CorTechs Labs, San Diego, CA, USA) that performs automatic volumetric measurements, providing percentile volume data of brain regions referenced against an age and gender-matched normative cohort.^12^

We hypothesize that subtle structural evidence of epileptic network pathology extending beyond the temporal lobe reduces the odds of seizure freedom after surgery. If this is correct, qMRI measured in the context of routine clinical practice could be leveraged to enhance individualized seizure outcome prediction prior to TLE surgery. Given the advanced analytical tools, the epilepsy community should explore the incorporation of volumetric measurements into routine clinical care.

## MATERIALS AND METHODS

### Patient selection

In this multicentre retrospective study, we selected patients who underwent temporal lobe surgery for epilepsy (*n* = 653). We excluded patients with multilobar resections (*n* = 86), prior brain surgeries (*n* = 44), post-operative events of unclear nature (*n* = 22), and patients who did not have an available pre-operative high-resolution 3D T1-weighted MRI (*n* = 66). The final cohort included 435 patients treated at Cleveland Clinic, USA (*n* = 289) Mayo Clinic, USA (*n* = 57) and University of Campinas, Brazil (*n* = 89) from 2010 to 2018.

Demographic and clinical data were collected from medical records. All patients underwent a comprehensive pre-surgical assessment, including clinical history and video-EEG, and with magnetoencephalography, nuclear imaging with fluorodeoxyglucose positron emission tomography and/or single-photon emission tomography when indicated. Visual MRI analysis was performed by a neuroradiologist specialized in epilepsy who was blinded to post-surgical seizure outcomes.

The following potential seizure outcome predictors were considered: pre-operative seizure frequency, age at epilepsy onset, age at surgery, duration of epilepsy, sex, aetiology, side of surgery, presence of generalized tonic-clonic seizures, MRI abnormalities and type of surgery. Aetiology was defined by pathology or MRI findings (when pathology results were not available). These outcome predictors were similar to the ones used in the generation of our already published epilepsy surgery nomogram.^5^

### Seizure outcomes

The primary outcome was defined by seizure control at last follow-up. Acute seizures were defined as seizures occurring within the first month after surgery and were not considered as seizure recurrence unless they persisted beyond the acute post-operative phase. Two separate analyses were done: one defining seizure control as complete postoperative seizure freedom; and one defining it as maintaining an Engel score^13^ of Ia or Ib (allowing for some postoperative seizures but eventual seizure control by the last follow-up).

### Quantitative MRI

The pre-operative 3D T1-weighted high-resolution MRIs were de-identified and sent to Neuroquant for quantitative analysis. The software calculates the volume of 71 brain regions providing the left, right and total volume of each region.

For these analyses, we excluded the following brain regions: brainstem, cerebellum, choroid plexus and individual ventricles (the total ventricular volume was included in the analysis), which resulted in 58 regions and intracranial volume measured in percentiles (a total of 175 measurements: left, right and asymmetry index). The percentile results compare the volumes of the different brain regions against an age and gender-matched normative cohort. NeuroQuant’s normative database is built on a population-based sample data set collected from several thousand subjects from 3 to 100 years of age with an equivalence of gender.

Neuroquant uses the percentage of intracranial volume difference between left and right volumes divided by the mean to calculate the asymmetry index. This value is then compared to the normative database, and results are provided in percentile. When interpreting asymmetry values as percentiles, the closer the value is to 50, the smaller the difference between left and right volumes. If the asymmetry value measured in percentile is between 1 and 49, the left side is smaller than right, and if the value is between 51 and 100, the right side is smaller.

We analysed right- and left-sided surgeries separately to account for structural and functional inter-hemispheric differences.^14^^,^^15^

#### Surgical Lacuna

To evaluate whether the ipsilateral temporal regions included in the models were resected or not, we reviewed all available post-operative MRIs (354 patients, including 144 on the right side and 210 on the left side). Anatomical sub-regions relevant to the model predictive performance were classified as being resected or not (partial resections were included in the resection group).

### Statistical analysis

#### Demographics and clinical data

Statistical analysis was run for the right side and the left side of the surgery separately. For each side of surgery, the patients’ collected information collected was summarized as the mean and standard deviation for continuous variables, and as counts and percentage for all categorical variables.

A two-sample *t-*test was performed for comparing continuous variables by outcomes, while categorical variables were analysed by the Chi-square test. Fisher’s Exact test was used when one or more of the cells had an expected frequency of five or less. The Bonferroni correction procedure was applied to account for multiple comparisons.

We used the complete-case analysis to address missing data.

#### qMRI: variable selection procedure

**Figure 1:**
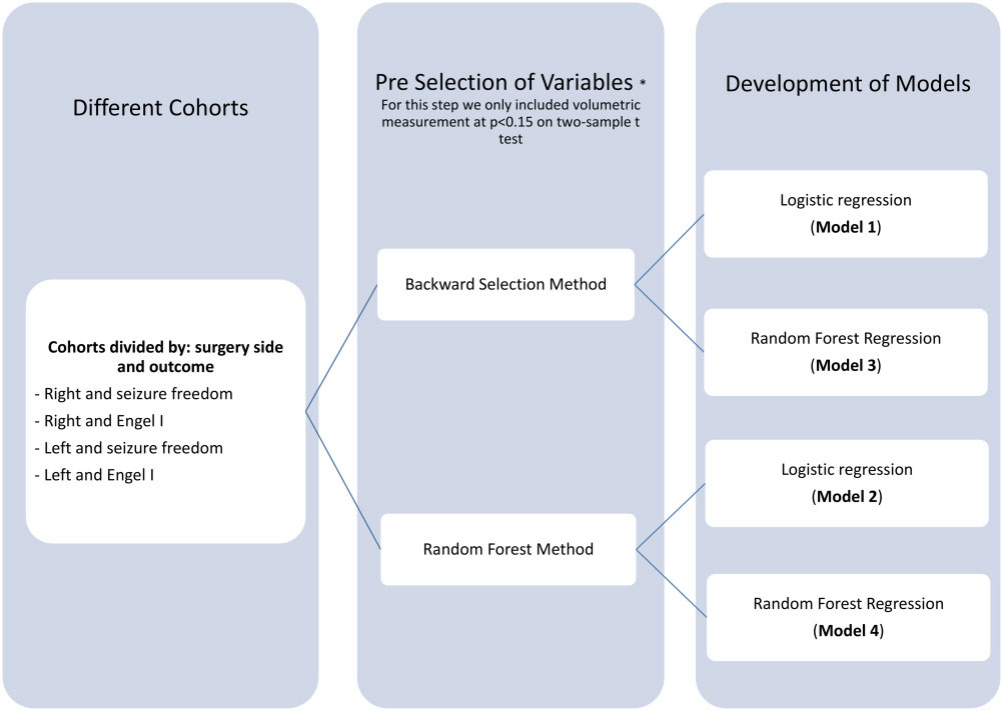
Statistical analysis: flowchart illustrating model development and process of model selection.

The statistical method used is illustrated in Fig. 1. Due to the high number of variables, a selection procedure was performed. For the qMRI analysis, we used the volume of different brain regions measured in percentiles. Variables at *P* < 0.15 on a two-sample *t*-test were pre-selected as potential predictor variables. Correlation analysis of the predictors was conducted to avoid multi-collinearity in a regression model.

Both backward elimination using Akaike’s information criterion (AIC)^16^ as a selection criteria, and random forest selection methods were performed. For the backward elimination method, we started with a model that included all variables and calculated the AIC value. The AIC is a measure of the relative goodness of fit for a specific set of data, which is used to perform model comparisons. Lower AIC indicates a better model. By removing one variable at a time from the initial model, we created new models with new AIC values. The model with the lowest AIC was selected. The same procedure was then repeatedly performed in the newer models until we had a final model with the lowest AIC value. The variables selected in this model will be those used for the analysis. Random forest variable selection ranks explanatory (independent) variables using the random forest score of importance (i.e. large values are ranked more important than low values). Random forest computes how much each variable decreases the node impurity (e.g. potential for misclassification). The most important variable is the one that decreases the impurity the most. The final importance of a variable is the average of the impurity decrease for each variable across all the trees. Two sets of predictors were selected after the backward elimination method and random forest selection method.

#### Development of models to predict seizure outcome using clinical and qMRI data

To develop predictive models, we created four models applied to the right- and left-sided surgeries with outcomes being either seizure free or Engel I at last follow-up (Fig. 1).

- Model 1: we used the backward method to pre-select the variables and performed a logistic regression.
- Model 2: we used random forest to pre-select the variables and performed a logistic regression.
- Model 3: we pre-selected variables using the backward method and performed a random forest regression.
- Model 4: we pre-selected variables using a random forest selection method and performed a random forest regression.

Compared to the logistic regression, random forest regression does not assume the model has a linear relationship, and it utilizes ensemble learning. Random forest regression takes random samples, forms many decision trees, and then averages out the leaf nodes to get a more precise model. We selected these two modelling methodologies because logistic regression is the most classical prediction model methodology (a benchmark method) and random forest modelling has been shown to be one of the most reliable machine learning approaches outperforming other machine learning methods^17^ such as support vector machines^18–20^ when the sample size is moderate or small.

The concordance index of each model was calculated. The concordance index is used to compare the goodness of fit of logistic regression models.^3^

#### Development of models to predict seizure outcome using clinical data only

To evaluate the impact of qMRI on the model's performance, we also created models including only clinical predictors applied to the right- and left-sided surgeries with outcomes being either seizure free or Engel I at last follow-up. We used logistic regression and random forest regression to create the models and the concordance index to evaluate performance.

#### Testing dataset

The entire patient dataset served as both development and validation cohorts. To adjust the concordance index, the whole cohort was randomly split into training and testing sets, with 70% used for training and 30% for testing. Using the training dataset, we performed the original regression to the model outcome as a function of the predictors selected. The model's performance was assessed on the testing dataset. This process was repeated 100 times with different random seeds, and the mean and 95% confidence limit of the area under the curve was calculated.

We opted for the bootstrap method instead of the traditional k-fold method to account for the heterogeneity of the different cohorts regarding type of surgery and epilepsy aetiology.

#### Final model

The concordance index calculated for the testing dataset was used as a measure of the predictive accuracy of the model. The respective C-indices were compared to each other, and the model with the highest concordance index was selected as the final model.

All analyses were performed using R studio software. The level of statistical significance was set at *P* < 0.05 (two-tailed). To describe the cohort, we used the median (interquartile range) for numeric variables, and counts (%) for categorical data. Kruskal–Wallis and Fisher’s Exact tests were used to test for univariate associations of numeric and categorical variables with the treatment, respectively. Lastly, we analysed whether the presence of one or more extra-temporal abnormalities in the extra-temporal regions of interest identified by the final model correlated with seizure outcomes (we defined abnormality as volumes less than 5% compared to the normative population^21^^,^^22^).

#### Graphic visualization

For the graphic visualization of these results, the significant regions of interest included in the final models were identified on a 3D MRI brain atlas: Neuromorphometrics (http://www.neuromorphometrics.com/), and the *t*-values were displayed using a colour scale to highlight the selected areas, with cool colours representing negative *t*-values and hot colours positive *t*-values.

### Standard protocol approvals, registrations and patient consent

The Cleveland Clinic Institutional Review Board approved this study and waived the requirement for individual informed consent. All data from participating sites (Mayo Clinic, USA and University of Campinas, Brazil) were de-identified of all patient health information.

### Data availability statement

The data that support the findings of this study are available from the corresponding author, LJ, upon reasonable request.

## RESULTS

### Patient characteristics

A total of 435 patients were included in this cohort. Median follow-up time post-surgery was 34 months (25th/75th, 17/60) with a maximum follow-up of 116 months. Tables 1 and 2 display summary statistics for seizure freedom and Engel I outcomes at the last follow-up, respectively. The initially investigated variables are displayed in Tables 1 and 2.

**Table 1:** Summary statistics of the cohort (seizure-free versus not seizure-free outcome)

			Post-operative outcome				
	Overall		Not seizure-free		Seizure-free		
	(*N* = 435)		(*N* = 172)		(*N* = 263)		
Variables	*N*	Statistics	*n*	Statistics	*n*	Statistics	*P*-value*
Mean monthly pre-op sz freq	434	16.4 ± 34.8	171	18.0 ± 38.5	263	15.3 ± 32.3	0.84^a^
Age at epilepsy onset	435	18.2 ± 15.1	172	19.1 ± 15.4	263	17.6 ± 15.0	0.64^a^
Age at surgery	435	37.4 ± 15.2	172	38.6 ± 14.9	263	36.6 ± 15.4	0.36^a^
Epilepsy duration	435	19.3 ± 14.8	172	19.6 ± 15.4	263	19.0 ± 14.4	1.00^a^
Sex	435		172		263		1.00^b^
Female		218 (50.1)		89 (51.7)		129 (49.0)	
Male		217 (49.9)		83 (48.3)		134 (51.0)	
Aetiology	435		172		263		***<0.001^b^***
MCD		15 (3.4)		4 2.3)		11 (4.2)	
MTS		198 (45.5)		62 (36.0)		136 (51.7)	
Tumour		40 (9.2)		11 (6.4)		29 (11.0)	
Other		182 (41.8)		95 (55.2)		87 (33.1)	
Surgery side	435		172		263		1.00^b^
Left		246 (56.6)		99 (57.6)		147(55.9)	
Right		189 (43.4)		73 (42.4)		116(44.1)	
MRI	434		172		262		0.13^b^
Abnormal		361 (83.2)		136 (79.1)		225(85.9)	
Normal		73 (16.8)		36 (20.9)		37(14.1)	
Presence of GTC seizures	428		169		259		***0.008^b^***
No		82 (19.2)		21 (12.4)		61(23.6)	
Yes		346 (80.8)		148 (87.6)		198(76.4)	
Type of surgery	435		172		263		
AH		81 (18.6)		25 (14.5)		56 (21.3)	0.28^b^
Standard TL		264 (60.7)		106 (61.6)		158 (60.1)	
Sparing the HC		90 (20.7)		41 (23.8)		49 (21.3)	

Statistics presented as mean ± SD or *N* (column %); *P*-values.

a Two-sample *t*-test.

b Pearson's chi-square test.

AH = amygdalohippocampectomy; GTC = generalized tonic-clonic; HC = hippocampus; MCD = malformation of cortical development; MRI = magnetic resonance image; MTS = mesial temporal sclerosis; Pre-op sz freq = pre-operative seizure frequency; TL = temporal lobectomy.

* *P*-values are adjusted using the Bonferroni correction method.

**Table 2:** Summary statistics of the cohort (Engel I versus II–IV outcome)

			Post-operative outcome				
	Overall		**II**–**IV**		I		
	(*N* = 435)		(*N* = 134)		(*N* = 301)		
Factor	*N*	Statistics	*n*	Statistics	*n*	Statistics	*P*-value*
Mean monthly pre-op sz freq	434	16.4 ± 34.8	134	20.1 ± 42.0	300	14.7 ± 31.1	0.26^a^
Age at epilepsy onset	435	18.2 ± 15.1	134	19.1 ± 15.3	301	17.8 ± 15.1	0.80^a^
Age at surgery	435	37.4 ± 15.2	134	37.1 ± 15.2	301	37.5 ± 15.3	1.00^a^
Epilepsy duration	435	19.3 ± 14.8	134	18.1 ± 14.6	301	19.8 ± 14.8	0.52^a^
Sex	435		134		301		1.00^b^
Female		218 (50.1)		70 (52.2)		148 (49.2)	
Male		217 (49.9)		64 (47.8)		153 (50.8)	
Aetiology	435		134		301		***<0.001^b^***
MCD		15 (3.4)		3 (2.2)		12 (4.0)	
MTS		198 (45.5)		41 (30.6)		157 (52.2)	
Tumour		40 (9.2)		7 (5.2)		33 (11.0)	
Other		182 (41.8)		83 (61.9)		99 (32.9)	
Surgery side	435		134		301		1.00^b^
Left		246 (56.6)		78 (58.2)		168 (55.8)	
Right		189 (43.4)		56 (41.8)		133 (44.2)	
MRI	434		134		300		***0.018^b^***
Abnormal		361 (83.2)		102 (76.1)		259 (86.3)	
Normal		73 (16.8)		32 (23.9)		41 (13.7)	
Presence of GTC seizures	428		131		297		0.12^c^
No		82 (19.2)		18 (13.7)		64 (21.5)	
Yes		346 (80.8)		113 (86.3)		233 (78.5)	
Type of surgery	435		134		301		***0.004^c^***
AH		81 (18.62)		14 (10.45)		67 (22.26)	
Standard TL		264 (60.69)		82 (61.19)		182 (60.47)	
Sparing the HC		90 (20.69)		38 (28.36)		52 (17.28)	

Statistics presented as mean ± SD or *N* (column %); *P*-values

a Two-sample *t*-test.

b Pearson's chi-square test.

AH = amygdalohippocampectomy; GTC = generalized tonic-clonic; HC = hippocampus; MCD = malformation of cortical development; MRI = magnetic resonance image; MTS = mesial temporal sclerosis; Pre-op sz freq = pre-operative seizure frequency; TL = temporal lobectomy.

* *P*-values are adjusted using the Bonferroni correction method.

### Comparing different models

We used different techniques to create models to predict the probability of being seizure-free and the probability of an Engel I at last follow-up according to the side of surgery. Models including only clinical variables were also created for comparison. Table 3 displays the c-indices of these different models. After comparing the respective c-indices to each other, we selected Model 1 as our final model: The logistic regression model, including clinical variables using the backward elimination method as a selection procedure (adjusted concordance index) (Table 3).

**Table 3:** Comparison between different models

		qMRI + clinical predictors		Clinical predictors only	
Description	Model	Full Model C-index (CI)	Adjusted C-index (CI)	Full model C-index (CI)	Adjusted C-index (CI)
Seizure free (yes versus no) right-sided model	1	0.829 (0.77–0.89)	0.703 (0.69–0.72)	0.672 (0.59–0.75) (logistic regression)	0.582 (0.57–0.59)
	2	0.788 (0.72–0.86)	0.659 (0.65–0.67)		
	3	0.701 (0.62–0.78)	0.675 (0.66–0.69)	0.598 (0.52–0.68) (random forest regression)	0.586 (0.58–0.60)
	4	0.710 (0.63–0.79)	0.689 (0.68–0.70)		
Seizure free (yes versus no) left-sided model	1	0.762 (0.70–0.82)	0.664 (065–0.68)	0.680 (0.61–0.75) (logistic regression)	0.605 (0.59–0.62)
	2	0.740 (0.68–0.80)	0.642 (0.63–0.65)		
	3	0.660 (0.59–0.73)	0.647 (0.64–0.66)	0.574 (0.50–0.65) (random forest regression)	0.571 (0.56–0.58)
	4	0.616 (0.54–0.69)	0.614 (0.60–0.61)		
Engel I versus II–IV right-sided model	1	0.784 (0.71–0.86)	0.666 (0.65–0.68)	0.701 (0.62–0.78) (logistic regression)	0.623 (0.61–0.64)
	2	0.723 (0.64–0.81)	0.630 (0.61–0.65)		
	3	0.594 (0.50–0.69)	0.588 (0.57–0.60)	0.620 (0.54–0.71) (random forest regression)	0.594 (0.58–0.61)
	4	0.619 (0.53–0.71)	0.611 (0.60–0.62)		
Engel I versus II–IV left-sided model	1	0.821 (0.77–0.88)	0.729 (0.72–0.74)	0.733 (0.67–0.80) (logistic regression)	0.676 (0.67–0.69)
	2	0.813 (0.76–0.87)	0.722 (0.71–0.73)		
	3	0.701 (0.63–0.78)	0.686 (0.68–0.70)	0.654 (0.58–0.73) (random forest regression)	0.651 (0.64–0.66)
	4	0.727 (0.66–0.80)	0.715 (0.71–0.72)		

Adjusted C-index: concordance index adjusted using a random split method.

Models

1. Backward selection method, AIC as selection criteria—Logistic regression using selected variables.

2. Random Forest selection method—Logistic regression using selected variables.

3. Backward selection method—Random Forest regression using selected variables.

4. Random Forest selection method—Random Forest regression using selected variables.

CI = 95% confidence intervals.

When we evaluated models with ‘clinical predictors only’, we chose those created using a logistic regression as the final model based on the overall c-indices.

The 95% confidence intervals displayed in Table 3 demonstrate that the adjusted c-indexes from the final models with and without qMRI data (highlighted in grey) were significantly different.

**Figure 2:**
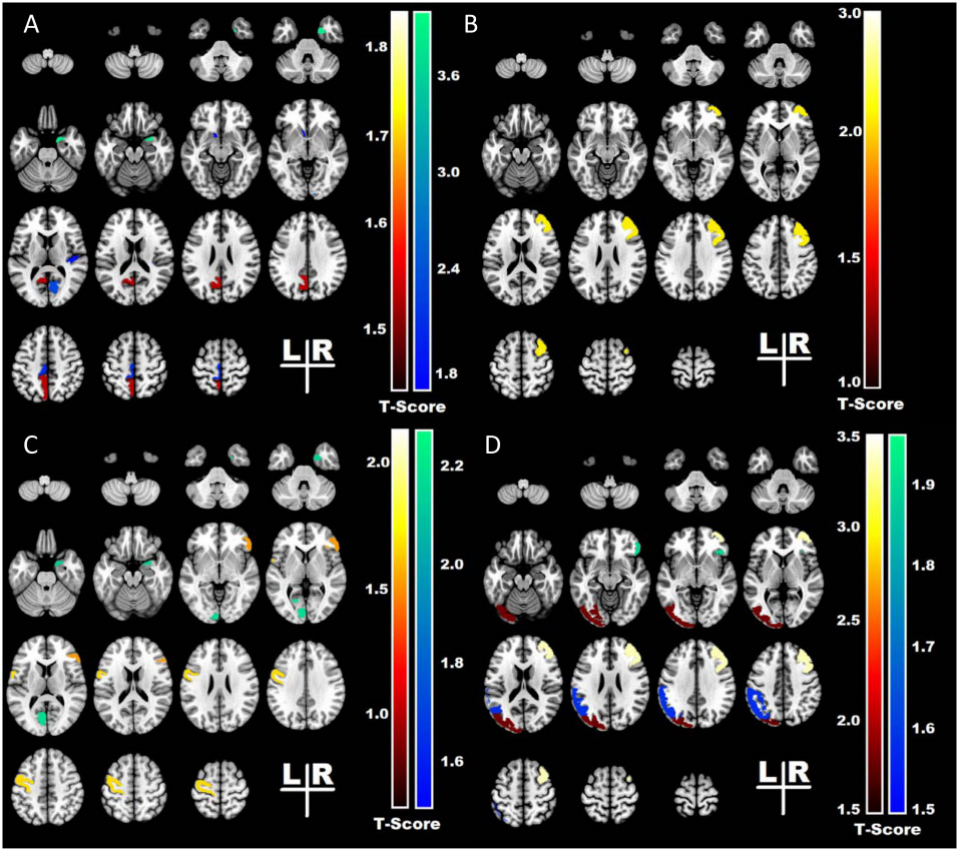
The heatmap represents the t statistics* of the univariate analysis and displays the structures that were identified by the model as outcome predictors. (**A**) Failure of postoperative seizure control for right-sided temporal lobe surgeries associated with smaller volumes of the transverse temporal, pericalcarine and entorhinal cortex in the right (ipsilateral) hemisphere, and with larger volumes of medial parietal in the left (contralateral) hemisphere. Seizure recurrence also associated with asymmetry of nucleus accumbens and paracentral regions, with the left side (contralateral hemisphere) smaller than the right. (**B**) Failure of postoperative seizure control for left-sided temporal lobe surgeries associated with smaller volumes of the right middle frontal region in the right (contralateral) hemisphere, and with asymmetry of middle frontal gyri, with the right side (contralateral hemisphere) smaller than the left. (**C**) Worse outcomes (Engel II–IV) for right-sided temporal lobe surgeries associated with smaller volumes of the entorhinal cortex in the right (ipsilateral) hemisphere, and with smaller volumes of the pericalcarine cortex and with larger volumes of the primary motor cortex in the left (contralateral) hemisphere. (**D**) Worse outcomes (Engel II–IV) for left-sided temporal lobe surgeries associated with smaller volumes of the pars orbitalis in the right (contralateral) hemisphere and asymmetry of the occipital lobe, and inferior parietal region with the left side (ipsilateral) hemisphere smaller than the right and asymmetry of middle frontal region with the right side (contralateral hemisphere) smaller than the left. * t-statistics represented in colour bars: cool colours negative t-values and hot colours positive t values.

The variables identified by the final models as outcome predictors are displayed in Tables 4 and 5. The graphic visualization of these results is shown in Fig. 2.

**Table 4:** Right-sided surgeries and predictors of surgical outcome

	Variables included in the model		Seizure free (yes versus no)				Engel I versus II-IV			
			Adjusted C: 0.703				Adjusted C: 0.666			
			Not sz free	sz free	*P*-value	coef	**Engel II**–**IV**	Engel I	*P*-value	coef
			(*n* = 73)	(*n* = 116)			(*n* = 56)	(*n* = 133)		
Clinical variables	Preop sz freq		18.1 ± 38.3	10.6 ± 19.5	0.08	−0.014	21.0 ± 42.9	10.3 ± 18.5	0.01	−0.022
	Gender	Male	33(45.2)	57(49.1)	0.56	0.226				
	Epilepsy duration		21.3 ± 15.4	19.1 ± 13.6	0.16	−0.023				
	GTCS	Yes	63(88.7)	92(80.7)	0.05	−1.114	47(87.0)	108(82.4)	0.52	−0.353
	Type of surgery	Sparing	12 (16.44)	10 (8.26)	0.14	−1.362	11 (19.64)	11 (8.27)	0.009	−2.292
		Resection	50 (68.49)	85 (73.28)	0.38	−0.531	38 (67.86)	97 (72.93)	0.09	−1.043
	MRI	Normal	15 (20.55)	18 (15.65)	0.58	0.323	13 (23.21)	20 (15.15)	0.58	0.31
	Etiology	MTS	27(37.0)	62(53.4)	0.46	−0.688	20(35.7)	69(51.9)	0.15	−1.350
		Tumour	6(8.2)	11(9.5)	0.56	−1.767	4(7.1)	13(9.8)	0.75	−0.360
		Other	36(49.3)	36(31.0)	0.06	−0.620	29(51.8)	43(32.3)	0.05	−1.870
**qMRI-specific regions**			**Mean volume (percentile)**				**Mean volume (percentile)**			
Right hemispheric regions	Right parahippocampal		25.1 ± 24.1	32.7 ± 29.4	0.20	0.010				
	Right middle temporal		26.0 ± 27.6	33.0 ± 30.0	0.18	0.010				
	Right transverse temporal		52.7 ± 33.9	60.4 ± 30.1	**0.02**	0.014				
	Right pericalcarine		53.6 ± 31.0	63.6 ± 29.8	**0.03**	0.014				
	Right entorhinal cortex		27.7 ± 30.7	47.1 ± 36.0	**0.02**	0.013	31.4 ± 33.0	43.1 ± 35.8	**0.03**	0.012
	Right inferior frontal						37.5 ± 30.6	30.5 ± 27.4	0.21	−0.009
Left hemispheric regions	Left medial parietal		66.5 ± 27.7	59.4 ± 31.3	**0.02**	−0.016				
	Left rostral anterior cingulate		59.4 ± 28.1	51.8 ± 29.0	0.14	−0.011	61.1 ± 28.4	52.0 ± 28.6	0.054	−0.014
	Left pericalcarine						48.5 ± 34.0	59.4 ± 30.4	**0.03**	0.014
	Left primary motor						49.6 ± 32.7	40.4 ± 32.5	**0.004**	−0.019
**qMRI-asymmetry index**			**Mean percentile**				**Mean percentile**			
Asym index percentile	Isthmus cingulate Asym		46.2 ± 34.2	54.5 ± 31.4	0.07	0.012				
	Nucleus accumbens Asym		42.5 ± 33.7	50.5 ± 33.3	**0.004**	0.018				
	Paracentral Asym		45.2 ± 33.6	54.0 ± 29.4	**0.004**	0.021				
	Superior parietal Asym						55.7 ± 33.8	64.2 ± 29.8	0.056	0.012

Statistics presented as mean ± SD or *N* (column %).

Asym = asymmetry; coef = model's coefficient; GTC = generalized tonic-clonic seizures; MTS = mesial temporal sclerosis; Pre-op sz freq = pre-operative seizure frequency; *P*-values = model's *P*-value; qMRI = quantitative magnetic resonance image.

**Table 5:** Left-sided surgeries and predictors of surgical outcome

	Variables included in the model		Seizure free (yes versus no)				Engel I versus II–IV			
			Adjusted C: 0.664				Adjusted C: 0.729			
			**Not sz free** (*n* = 99)	**sz free** (*n* = 147)	*P*-value	coef	Engel II–IV (*n* = 78)	**Engel I** (*n* = 168)	*P*-value	coef
Clinical variables	Preop sz freq		18.0 ± 38.8	18.9 ± 39.3	0.90	0.001	19.5 ± 41.6	18.1 ± 37.8	0.67	−0.002
	Gender	Male	50(50.5)	77(52.4)	0.63	−0.154				
	Epilepsy duration		18.3 ± 15.2	19.0 ± 15.0	0.92	−0.001				
	GTCS	Yes	85(86.7)	106(73.1)	**0.01**	−0.070	66(85.7)	125(75.3)	0.08	−0.815
	Type of surgery	Sparing	29 (29.29)	39 (26.53)	0.09	1.161	27 (34.62)	41 (24.40)	0.59	−0.415
		Resection	56 (56.57)	73 (49.66)	0.35	0.528	44 (56.41)	85 (50.60)	0.48	−0.476
	MRI	Normal	21 (21.21)	19 (12.93)	0.56	0.263	19 (24.36)	21 (12.50)	0.44	0.385
	Aetiology	MTS	35(35.4)	74(50.3)	0.82	−6.913	21(26.9)	88(52.4)	0.80	−7.934
		Tumor	5(5.1)	18(12.2)	0.81	−7.350	3(3.8)	20(11.9)	0.82	−7.165
		Other	59(59.6)	51(34.7)	0.79	−8.407	54(69.2)	56(33.3)	0.76	−9.408
qMRI-specific regions			Mean volume (percentile)				Mean volume (percentile)			
Right hemispheric regions	Right middle frontal		45.5 ± 29.4	56.5 ± 29.1	**0.03**	0.017				
	Right premotor		50.0 ± 29.5	56.2 ± 29.1	0.17	−0.011				
	Right parahippocampal		32.6 ± 29.1	39.4 ± 30.2	0.07	0.010	32.0 ± 27.8	38.9 ± 30.7	0.11	0.010
	Right putamen		67.5 ± 27.2	60.4 ± 30.6	0.11	−0.009	69.4 ± 25.5	60.4 ± 30.8	0.11	−0.010
	Right pars orbitalis						34.4 ± 31.9	42.3 ± 30.2	**0.02**	0.014
Left hemispheric regions	Left pallidum		15.1 ± 23.0	23.9 ± 29.7	0.08	0.013	63.9 ± 27.1	56.3 ± 31.6	0.72	0.003
	Left lateral orbitofrontal						53.9 ± 33.3	45.1 ± 32.5	0.39	−0.005
	Left transverse temporal						55.6 ± 31.3	61.6 ± 29.7	0.06	0.011
qMRI-asymmetry index			Mean percentile				Mean percentile			
Asymmetry index percentile	Anterior cingulate Asym		59.5 ± 29.2	51.9 ± 32.0	0.18	−0.009				
	Ventral diencephalon Asym		35.4 ± 32.3	41.8 ± 30.8	0.07	0.009				
	Middle frontal Asym		59.2 ± 29.3	49.5 ± 30.9	**0.03**	0.017	62.8 ± 27.9	49.0 ± 30.9	**0.004**	−0.017
	Medial parietal Asym						37.9 ± 31.4	45.7 ± 33.4	0.11	0.009
	Occipital lobe Asym						44.9 ± 29.3	37.7 ± 29.1	**0.02**	−0.014
	Inferior parietal Asym						41.3 ± 29.1	48.0 ± 31.7	**0.04**	0.012
	CWMH Asym						42.4 ± 31.6	51.3 ± 35.1	**0.05**	0.010

Statistics presented as mean ± SD or *N* (column %). Bolded p-values are statistically significant.

Asym = asymmetry; coef = model's coefficient; CWMH = cerebral white matter hypointensities; GTC = generalized tonic-clonic seizures; MTS = mesial temporal sclerosis; Pre-op sz freq = pre-operative seizure frequency; *P*-values: model's *P*-value; qMRI = quantitative magnetic resonance image.

### Predictors of surgical outcome in RIGHT-sided surgeries

#### Complete seizure freedom versus seizure recurrence

In patients with right-sided temporal lobe resection, smaller cortical volumes in the ipsilateral transverse temporal (*P* = 0.021), entorhinal (*P* = 0.021) and pericalcarine cortices (*P* = 0.029), and larger volumes in the contralateral parietal region (*P* = 0.019) were associated with failure of postoperative seizure freedom (Fig. 2A).

When evaluating asymmetry findings, the group with recurrent seizures had smaller volumes in the contralateral nucleus accumbens (*P* = 0.004) and paracentral region (*P* = 0.04) (Table 4).

#### Engel I seizure outcome versus Engel II–IV

In the right-sided surgery group, a higher pre-operative seizure frequency (*P* = 0.014), surgery sparing the hippocampus (*P* = 0.009) and aetiology: other (p0.045) predicted Engel II–IV outcome. Smaller volumes of the ipsilateral entorhinal (*P* = 0.033) and contralateral pericalcarine cortex (*P* = 0.026), larger volumes of the contralateral primary motor area (*P* = 0.004) (Fig. 2B) also predicted Engel II–IV outcomes (Table 4).

### Predictors of the surgical outcome in LEFT-sided surgeries

The areas included in the model were different when surgery was performed on the left side.

#### Complete seizure freedom versus seizure recurrence

In patients with left-sided temporal lobe resection, history of generalized tonic-clonic seizures (*P* = 0.011), smaller cortical volumes in the contralateral middle frontal region (*P* = 0.033) and the degree of asymmetry in the middle frontal region (*P* = 0.033), with the contralateral side being smaller, predicted seizure recurrence (Fig. 2C).

#### Engel I seizure outcome versus Engel II–IV

In the left-sided surgery group, predictors of Engel II**–**IV were smaller volumes of the contralateral pars orbitalis (*P* = 0.016) (Fig. 2D), asymmetry of the middle frontal region, with the side contralateral to the surgery smaller (*P* = 0.004), asymmetry of the occipital lobe (*P* = 0.023), inferior parietal region (*P* = 0.042) and cerebral white matter hypointensities (*P* = 0.046), with the ipsilateral side being smaller (Table 5).

#### Surgical Lacuna

In the left-sided surgery group, the ipsilateral transverse temporal gyrus was the only temporal region included in the model to predict Engel I outcome. Only 9/210 (4.3%) had that structure resected or partially resected. In the right-sided surgery group, the percentage of patients who had the following ipsilateral temporal regions resected is as follows: parahippocampal gyrus (133/144, 92.4%), entorhinal cortex (133/144, 92.4%) middle temporal gyrus (129/144, 89.6%) and transverse temporal gyrus (8/144, 5.6%).

We then evaluated whether the resection of these areas was associated with surgical outcome, none was (Table 6).

**Table 6:** Seizure outcome and resection of the ipsilateral temporal regions included in the model

Variables included in the model		Surgical outcome		
		Resected/partially resected		
qMRI-specific regions		**Not sz free** (*n* = 55)	**sz free** (*n* = 89)	*P*-**value**
Right sided-surgery	Right parahippocampal	49/55 (89.1%)	84/89 (94.4%)	0.33^a^
	Right middle temporal	48/55 (87.3%)	81/89 (94.4%)	0.58^a^
	Right transverse temporal	6/55 (10.9%)	2/89 (2.25%)	0.54^a^
	Right entorhinal cortex	49/55 (89.1%)	84/89 (94.4%)	0.33^a^
qMRI-specific regions		Engel II–IV (*n* = 68)	Engel I (*n* = 142)	*P*-value
Left sided-surgery	Left transverse temporal	4/68 (6.9%)	5/142 (3.5%)	0.48^a^

Statistics presented as *N* (column %); *P*-values.

a Fisher exact test.

qMRI = quantitative MRI; sz = seizure.

#### Extra-temporal volume abnormalities

56.6% of patients with one or more abnormal regions of interest (bolded in Tables 4 and 5) were seizure-free at last follow-up as opposed to 68.0% of those with no abnormalities in any of these extra-temporal regions (*P*-value = 0.02).

## DISCUSSION

Despite significant advances in the evaluation of patients undergoing pre-surgical evaluation, our ability to predict surgical outcomes remains suboptimal.^3^^,^^4^ In the present study, we investigated if qMRI measurements could help develop a model to predict seizure outcome after TLE surgery.^5^

### The model

Since there is no clear consensus on the best statistical method to apply in the development of tools to predict outcomes, we compared models using the combination of two different selection methods (backward and random forest) and two different regressions (logistic regression and random forest). The logistic regression using the backward elimination method as a selection procedure had the highest c-statistics and was selected as the final model (Table 3). One of the strengths of this study is that we not only compared the importance of the quantitative and clinical variables in predicting surgical outcome but also explored different methods of conducting the statistical analysis.

We studied right- and left-sided surgeries separately to account for structural and functional interhemispheric differences.^14^^,^^15^ Studies using different neuroimaging techniques demonstrated that left TLE is usually associated with more diffuse and widespread changes compared to right TLE.^6^^,^^15^^,^^23^^,^^24^ In our study, volume differences were better predictors of outcome on the right-sided models compared to the left-sided, where asymmetry differences seemed to be better predictors. The ‘floor effect’ could explain this difference. Since the right-sided TLE seems to be a more unilateral disease, the presence of volume differences as predictors stands out as compared to left-sided TLE. Another possible contributor is the younger age at onset of epilepsy in left TLE as compared to the right TLE as demonstrated in a large multicentre study.^6^

If the left and right TLE groups were similar, we would expect a mirror effect, with the same structures being identified by the model. The fact that the structures were different reinforces the hypothesis that left and right TLE behave differently. Our results are, therefore, in agreement with the literature suggesting the left- and right-sided TLE should be viewed as aetiological and pathologically distinct entities with distinct outcome predictors.^14^^,^^15^^,^^23^^,^^25^ We should also consider the possibility that the lack of mirroring effect could be artificial, due to overfitting of the lateralized models or under powering thus failing to detect the same signals in each model. A large sample size is always intuitively desired for classification or regression studies, and a larger sample size theoretically can minimize the empirical risk. Cui and Gong^26^ conducted a comprehensive study on sample size effects for building prediction models in neuroimage studies. They showed greater improvements in the accuracy and stability of the prediction when the sample size is increased from an initially small sample size, whereas smaller improvements are observed when the sample size is increased from an initially large sample size. According to their findings, the average accuracy and stability of the prediction appear to plateau at sample sizes of 200–300, regardless of the algorithm. Therefore, a minimum sample size of 200 is recommended for machine learning regression prediction. Our study included 435 subjects, and the samples for building each of the sub-models were around 200. Even though we always desire larger sample sizes, compared to prior studies, we describe a cohort with a relatively reasonable size. Our sample may not fully represent the entire spectrum of the population, therefore limiting the generalizability of the predicted results to certain independent sample sets.

By adding the qMRI data to the model, we were able to increase the c-statistics further from 0.58 to 0.70 (right-sided surgery) and from 0.61 to 0.66 (left-sided surgery) for complete seizure freedom. For Engel I score prediction, the C-statistics increased from 0.62 to 0.67 (right-sided surgery) and from 0.68 to 0.73 (left-sided surgery). The models created in this study using clinical predictors only had similar c-statistics values compared to our previously published nomogram (the C-statistics for complete seizure freedom was 0.6, and for Engel I score 0.61). Even though the increase in the C-index was statistically significant, assessing the clinical significance of this enhancement is a nuanced exercise. Similar studies in other fields considered comparable c-statistic enhancements as an improvement in the model's performance. In lung cancer research, Mayo Clinic’s Solitary Pulmonary Nodule Malignancy Risk model was the well-known benchmark model for lung cancer prediction. Reid et al. developed improved models to help characterize Pulmonary Nodules considered high enough risk by a clinician to recommend a biopsy. In an independent sample used for validation, c-index for Reid’s model was 0.67 compared with 0.63 for the Mayo Clinic model, and is preferred as offering improved clinical utility.^27^ We believe any enhancement in the model's performance is important, especially considering the resources needed to generate this increase in the c-statistic in our study are relatively minor: Neuroquant is commercially available, Food and Drug Administration approved, and user friendly, so implementing it does not necessitate the typical major investments required to build a research imaging program within an epilepsy surgery centre.

Given the availability of clinical software packages for automated volume segmentation and measurements, our study provides now a new tool that could be incorporated in routine clinical practice to enhance surgical outcome prediction.

#### Regions of interest

Volumetric differences located predominantly in the ipsi- and contra-lateral fronto-central and temporal regions were associated with worse outcomes. The comparison between regions included in our model with that described in the literature revealed some interesting similarities. The Enhancing Neuro Imaging Genetics through Meta-Analysis-epilepsy study compared patients with epilepsy and controls, looking for areas with reduced volume ^6^ using freesurfer. Even though Neuroquant is a different tool, its analysis procedure is similar to the one performed by freesurfer with comparable results.^28^ Even though the Enhancing Neuro Imaging Genetics through Meta-Analysis-epilepsy study aimed to look for brain regions related to epilepsy regardless of seizure outcome, in general, many areas identified by our model as outcome predictors overlap with the ones reported by Enhancing Neuro Imaging Genetics through Meta-Analysis-epilepsy. The areas also overlap with other studies that reported progressive atrophy in the ipsilateral temporopolar and central regions and contralateral orbitofrontal, insular, and angular regions in TLE.^29^ A more rapid progression of atrophy was seen in the frontocentral and parietal regions in patients with longer duration of disease.^29^

Studies using similar morphometric techniques, including voxel-based morphometry, surface-shape analysis and cortical thickness, also reported an association between surgical outcome and morphometric changes in extrahippocampal structures^8^ like the entorhinal cortex,^30^ temporopolar and insular cortices,^11^ parahippocampal region,^31^ thalamotemporal structure^32^ and whole-brain extrahippocampal structures.^10^^,^^33^

Given many regions identified by our model as outcome predictors overlap with regions described in previous studies rejects the assumption that these areas might have been selected by chance. Our findings reinforce the involvement of these areas in the pathophysiology of TLE, explaining their relevance in predicting surgical outcome. Resections of the ipsilateral temporal lobe structures identified by this model were not associated with seizure outcome. A limitation of this sub-analysis was the uneven distribution of cases, with some of the evaluated structures being routinely removed and others rarely resected. For example, some structures, like the transverse gyrus, are rarely resected, making it difficult to analyse the role of this region in surgical outcomes. This limitation might have influenced our results, explaining why, even though these variables were predictive of outcome in the model, they were not associated with overall seizure freedom in the surgical lacuna analysis. Future studies focusing on the exact extent of the resection are needed to better address this relationship. Because we do not acquire post-operative MRIs routinely, many patients had missing data. This limitation might have led to a selection bias that needs to be considered when evaluating the results.

One unexpected result was the fact that the ipsilateral hippocampal volume had no predictive value for temporal lobe surgery outcome. One explanation could be the floor effect, in other words, since the ipsilateral hippocampus was already atrophic, the range of volume variability was limited restricting our ability to differentiate subgroups. If we analyse the subgroup of patients with hippocampal sclerosis, the median hippocampal volume percentile (which could range from 1 to 100) was 1 (interquartile range 1–11) on the left-sided surgery group and 1 interquartile range (1–12) on the right-sided group, confirming the floor effect. Even though unexpected, this finding is in agreement with other studies that could not find the association between hippocampal volume and surgical outcome.^8^ Furthermore, because the qMRI data were evaluated in conjunction with clinical data, we hypothesize that the presence of hippocampal sclerosis might have removed the additional information that hippocampal volumes might have provided.

### Epilepsy network as an outcome predictor

We tried to delineate the weight of each brain region included in the model in the definition of surgical results. However, we could not find a linear relationship. Instead, we found a more widespread pattern of atrophy, including areas that are likely important for outcome prediction, independent of the reduced cortical volumes being ipsi- or contra-lateral to the surgical site. The broad distribution of these regions of interest within and outside of the temporal lobe supports the current notion that focal epilepsies are in fact focal network disorders^7^^,^^23^^,^^34–36^ and the notion that the presence of damage and dysfunction outside of the surgical focus is relevant for the outcome.^6^^,^^29^ Volumetric differences in regions outside the surgical site probably mirror changes in the epileptic network and could work, for that reason, as a biomarker for surgical outcome.^37^ The correlation between the regions selected by the model and seizure outcome showed that patients with one or more abnormal regions had better outcomes, reinforcing the hypothesis that the more abnormal the network, the worse the outcome. Further studies will be necessary to define the exact functional connection between these areas.

### Clinical translation

Different techniques have been applied to several neuroimaging applications to improve the accuracy of predictive models.^38^ However, the translation from research findings into clinical care still requires improvement. We chose Neuroquant as a tool for MRI volumetry as it is Food and Drug Administration approved, clinically available, and practical. It performs an automated analysis, has a user-friendly interface, and promptly provides the volumetric results. Another benefit of Neuroquant is that it accepts high-resolution 3D images acquired using different MRI protocols. One limitation of this technique is that the software is not free of charge.

The novelty of this work is that the development of this preliminary model moves us one step forward towards the translation of research findings into a possible clinically meaningful and practical tool to be used by any institution, and not only by dedicated research centres. Although our findings need further confirmation, our results are robust. The data come from three different epilepsy centres, and our findings are consistent with previously published results. Our results also emphasize the need for statistical models to account for the complex relationship between quantitative measurement and surgical outcomes, and encourage the use of qMRI measurement to enhance our ability to predict surgical outcomes.

### Study limitations

The initial inclusion of most regions of interest provided by Neuroquant is both a limitation and an advantage. The limitation is that we included, at first, a high number of regions of interest. To overcome this issue, we performed a pre-selection of the most important predictive variables before the construction of the model. By doing so, we avoided a selection bias.

The creation of an online risk calculator would facilitate the introduction of our findings into clinical practice. However, the development of clinically useful calculators, based on models with multiple and complex variables, is challenging. Our results show that quantitative data can be used to improve the prediction of surgical outcome, however, new studies focused on automatic import of big data, visualization tools and innovative prediction methods are necessary to improve the usefulness of this tool. Further improvements are needed for this tool to be ready for general clinical deployment, such as additional external validation, and applications in diverse epileptic pathologies.

Even though we are comparing this model’s performance to the previously published nomogram’s,^5^ it is important to highlight that our study included only patients with temporal lobe resection, while the nomogram also included extra-temporal cases (32% of the development cohort).

Since we use regression models with a multitude of features, there is a potential for overfitting when calculating the full model c-index. To overcome this issue, we use the adjusted c-index.

## Conclusion

Our study demonstrates the prognostic value of qMRI in the context of brain surgery for drug-resistant TLE and provides a path for translation of sophisticated epilepsy imaging research into clinical application. The MRI volumetric measurements likely represent an intermediate quantitative trait that influences the surgical result. It is likely not the individual variable *per se* that defines the outcome, but the interplay among them. The volume of each region of interest may be a deconstruction of the endophenotype MRI,^39^ meaning that even though we assessed the areas selected by the model, the interpretation of the individual regions of interest should be done with caution. Regardless of the reason, the combination of variables ended up enhancing the performance of the model compared to our previous Nomogram.^5^ The models presented here provide an individualized prediction of surgical outcome with potentially clinically meaningful use.

## Abbreviations

AIC =: Akaike’s information criterion
GTCS = generalized tonic-clonic seizures
qMRI =: quantitative MRI
TLE =: temporal lobe epilepsy

## References

[1] Wiebe S , Blume WT, Girvin JP, Eliasziw M. A randomized, controlled trial of surgery for temporal-lobe epilepsy. N Engl J Med. 2001;345:311–318. 11484687 10.1056/NEJM200108023450501

[2] Engel J , McDermott MP, Wiebe S, et al Early surgical therapy for drug-resistant temporal lobe epilepsy: A randomized trial. JAMA - J Am Med Assoc. 2012;307:922–930. 10.1001/jama.2012.220 PMC4821633 22396514

[3] Gracia CG , Chagin K, Kattan MW, et al Predicting seizure freedom after epilepsy surgery, a challenge in clinical practice. Epilepsy Behav. 2019;95:124–130. 31035104 10.1016/j.yebeh.2019.03.047 PMC6546523

[4] Uijl SG , Leijten FSS, Arends JBAM, Parra J, Van Huffelen AC, Moons KGM. Prognosis after temporal lobe epilepsy surgery: The value of combining predictors. Epilepsia. 2008;49:1317–1323. 18557776 10.1111/j.1528-1167.2008.01695.x

[5] Jehi L , Yardi R, Chagin K, et al Development and validation of nomograms to provide individualised predictions of seizure outcomes after epilepsy surgery: A retrospective analysis. Lancet Neurol. 2015;14:283–290. 25638640 10.1016/S1474-4422(14)70325-4

[6] Whelan CD , Altmann A, Botía JA, et al Structural brain abnormalities in the common epilepsies assessed in a worldwide ENIGMA study. Brain. 2018;141:391–408. doi:10.1093/brain/awx341 29365066 PMC5837616

[7] Jehi L. Outcomes of epilepsy surgery for epileptic networks. Epilepsy Curr. 2017;17:160–162. 28684949 10.5698/1535-7511.17.3.160 PMC5486424

[8] Bonilha L , Keller SS. Quantitative MRI in refractory temporal lobe epilepsy: Relationship with surgical outcomes. Quant Imaging Med Surg. 2015;5:204–224. 25853080 10.3978/j.issn.2223-4292.2015.01.01 PMC4379322

[9] Keller SS , Glenn GR, Weber B, et al Preoperative automated fibre quantification predicts postoperative seizure outcome in temporal lobe epilepsy. Brain. 2017;140:68–82. 28031219 10.1093/brain/aww280 PMC5226062

[10] Yasuda CL , Valise C, Saúde AV, et al Dynamic changes in white and gray matter volume are associated with outcome of surgical treatment in temporal lobe epilepsy. Neuroimage. 2010;49:71–79. 19683060 10.1016/j.neuroimage.2009.08.014

[11] Bernhardt BC , Bernasconi N, Concha L, Bernasconi A. Cortical thickness analysis in temporal lobe epilepsy: Reproducibility and relation to outcome. Neurology. 2010;74:1776–1784. 20513813 10.1212/WNL.0b013e3181e0f80a

[12] Brewer JB. Fully-automated volumetric MRI with normative ranges: Translation to clinical practice. Behav Neurol. 2009;21:21–28. 19847042 10.3233/BEN-2009-0226 PMC5444284

[13] Engel JJ. Surgical treatment of the epilepsies. Raven Press; New York, USA; 1987.

[14] Ahmadi ME , Hagler DJ, McDonald CR, et al Side matters: Diffusion tensor imaging tractography in left and right temporal lobe epilepsy. Am J Neuroradiol. 2009;30:1740–1747. 19509072 10.3174/ajnr.A1650 PMC2759860

[15] Keller SS , Schoene-Bake JC, Gerdes JS, Weber B, Deppe M. Concomitant fractional anisotropy and volumetric abnormalities in temporal lobe epilepsy: Cross-sectional evidence for progressive neurologic injury. PLoS One. 2012;7:e46791. 23071638 10.1371/journal.pone.0046791 PMC3469561

[16] Akaike H. Information theory and an extension of the maximum likelihood principle. In: Parzen E., Tanabe K., Kitagawa G. (eds) Selected Papers of Hirotugu Akaike. Springer Series in Statistics (Perspectives in Statistics). Springer, New York, NY. 1998. 10.1007/978-1-4612-1694-0_15

[17] Segal MR. Machine learning benchmarks and random forest regression. UCSF: Center for Bioinformatics and Molecular Biostatistics. 2004. Retrieved from https://escholarship.org/uc/item/35x3v9t4.

[18] Statnikov A , Wang L, Aliferis CF. A comprehensive comparison of random forests and support vector machines for microarray-based cancer classification. BMC Bioinformatics. 2008;9:319. 18647401 10.1186/1471-2105-9-319 PMC2492881

[19] Deist TM , Dankers FJWM, Valdes G, et al Machine learning algorithms for outcome prediction in (chemo)radiotherapy: An empirical comparison of classifiers. Med Phys. 2018;45:3449–3459. 29763967 10.1002/mp.12967 PMC6095141

[20] Speiser JL , Miller ME, Tooze J, Ip E. A comparison of random forest variable selection methods for classification prediction modeling. Expert Syst Appl. 2019;134:93–101. 32968335 10.1016/j.eswa.2019.05.028 PMC7508310

[21] Louis S , Morita-Sherman M, Jones S, et al Hippocampal sclerosis detection with neuroquant compared with neuroradiologists. Am J Neuroradiol. 2020;41:591–597. 32217554 10.3174/ajnr.A6454 PMC7144657

[22] Luo W , Airriess C, Albright J. The NeuroQuant normative database comparing individual brain structures. CorTechs Labs, Inc. 2015;1–8.

[23] de Campos BM , Coan AC, Lin Yasuda C, Casseb RF, Cendes F. Large-scale brain networks are distinctly affected in right and left mesial temporal lobe epilepsy. Hum Brain Mapp. 2016;37:3137–3152. 27133613 10.1002/hbm.23231 PMC5074272

[24] Bonilha L , Rorden C, Halford JJ, et al Asymmetrical extra-hippocampal grey matter loss related to hippocampal atrophy in patients with medial temporal lobe epilepsy. J Neurol Neurosurg Psychiatry. 2007;78:286–294. 17012334 10.1136/jnnp.2006.103994 PMC2117646

[25] Coan AC , Appenzeller S, Bonilha L, Li LM, Cendes F. Seizure frequency and lateralization affect progression of atrophy in temporal lobe epilepsy. Neurology. 2009;73:834- 842. 19752449 10.1212/WNL.0b013e3181b783dd

[26] Cui Z , Gong G. The effect of machine learning regression algorithms and sample size on individualized behavioral prediction with functional connectivity features. Neuroimage. 2018;178:622–637. 29870817 10.1016/j.neuroimage.2018.06.001

[27] Reid M , Choi HK, Han X, et al Development of a risk prediction model to estimate the probability of malignancy in pulmonary nodules being considered for biopsy. Chest. 2019;156:367–375. 30940455 10.1016/j.chest.2019.01.038

[28] Ochs AL , Ross DE, Zannoni MD, Abildskov TJ, Bigler ED; Alzheimer's Disease Neuroimaging Initiative. For the Alzheimer’s disease neuroimaging initiative. comparison of automated brain volume measures obtained with NeuroQuant^®^ and FreeSurfer. J Neuroimaging. 2015;25:721–727. 25727700 10.1111/jon.12229

[29] Bernhardt BC , Worsley KJ, Kim H, Evans AC, Bernasconi A, Bernasconi N. Longitudinal and cross-sectional analysis of atrophy in pharmacoresistant temporal lobe epilepsy. Neurology. 2009;72:1747–1754. 19246420 10.1212/01.wnl.0000345969.57574.f5 PMC2827310

[30] Bernhardt BC , Kim H, Bernasconi N. Patterns of subregional mesiotemporal disease progression in temporal lobe epilepsy. Neurology. 2013;81:1840–1847. 24142475 10.1212/01.wnl.0000436069.20513.92 PMC3821710

[31] Keller SS , Cresswell P, Denby C, et al Persistent seizures following left temporal lobe surgery are associated with posterior and bilateral structural and functional brain abnormalities. Epilepsy Res. 2007;74:131–139. 17412561 10.1016/j.eplepsyres.2007.02.005

[32] Keller SS , Richardson MP, O'Muircheartaigh J, Schoene-Bake J-C, Elger C, Weber B. Morphometric MRI alterations and postoperative seizure control in refractory temporal lobe epilepsy. Hum Brain Mapp. 2015;36:1637–1647. 25704244 10.1002/hbm.22722 PMC4415572

[33] Bernhardt BC , Chen Z, He Y, Evans AC, Bernasconi N. Graph-theoretical analysis reveals disrupted small-world organization of cortical thickness correlation networks in temporal lobe epilepsy. Cereb Cortex. 2011;21:2147–2157. 21330467 10.1093/cercor/bhq291

[34] Jehi LE. Functional connectivity in mesial temporal lobe epilepsy: A dynamic concept. Epilepsy Curr. 2012;12:238–240. 23447723 10.5698/1535-7511-12.6.238 PMC3577132

[35] Bernhardt BC , Hong S, Bernasconi A, Bernasconi N. Imaging structural and functional brain networks in temporal lobe epilepsy. Front Hum Neurosci. 2013;7:624. 24098281 10.3389/fnhum.2013.00624 PMC3787804

[36] Vaughan DN , Rayner G, Tailby C, Jackson GD. MRI-negative temporal lobe epilepsy: A network disorder of neocortical connectivity. Neurology. 2016;87:1934–1942. 27694267 10.1212/WNL.0000000000003289

[37] Pitkänen A , Löscher W, Vezzani A, et al Advances in the development of biomarkers for epilepsy. Lancet Neurol. 2016;15:843–856. 27302363 10.1016/S1474-4422(16)00112-5

[38] Munsell BC , Wee CY, Keller SS, et al Evaluation of machine learning algorithms for treatment outcome prediction in patients with epilepsy based on structural connectome data. Neuroimage. 2015;118:219–230. 26054876 10.1016/j.neuroimage.2015.06.008 PMC4701213

[39] Alhusaini S , Whelan CD, Sisodiya SM, Thompson PM. Quantitative magnetic resonance imaging traits as endophenotypes for genetic mapping in epilepsy. NeuroImage Clin. 2016;12:526–534. 27672556 10.1016/j.nicl.2016.09.005 PMC5030372

